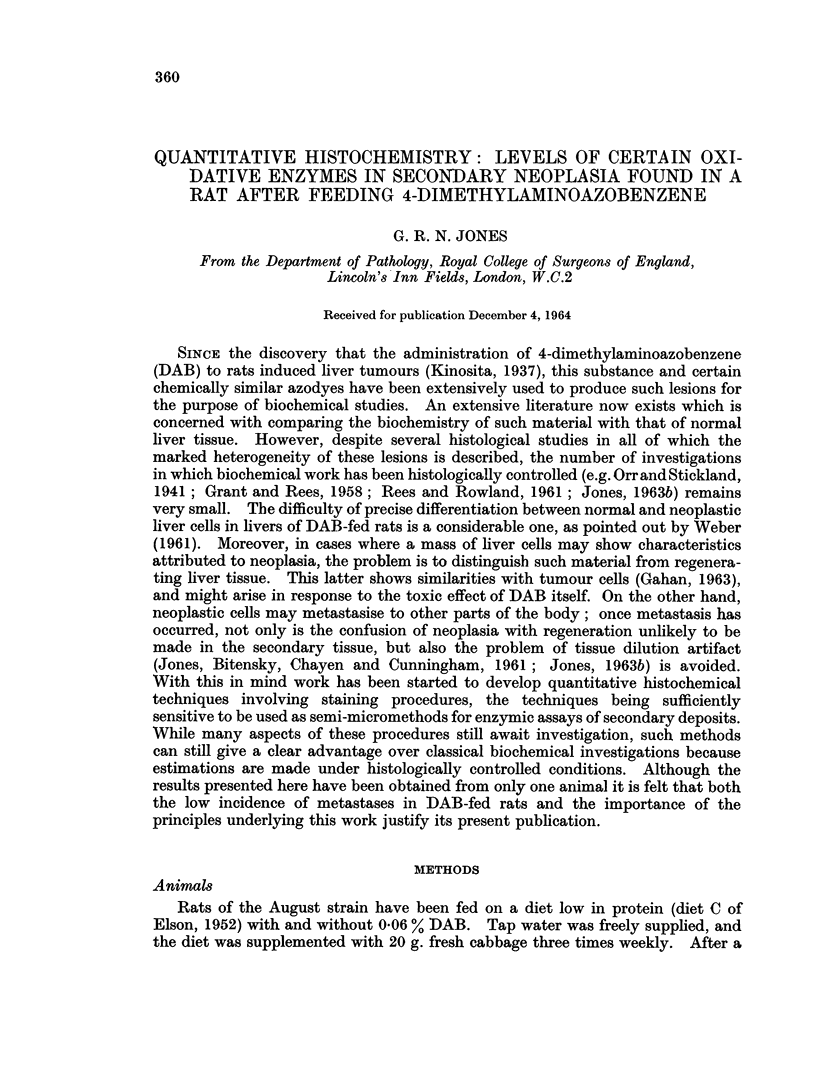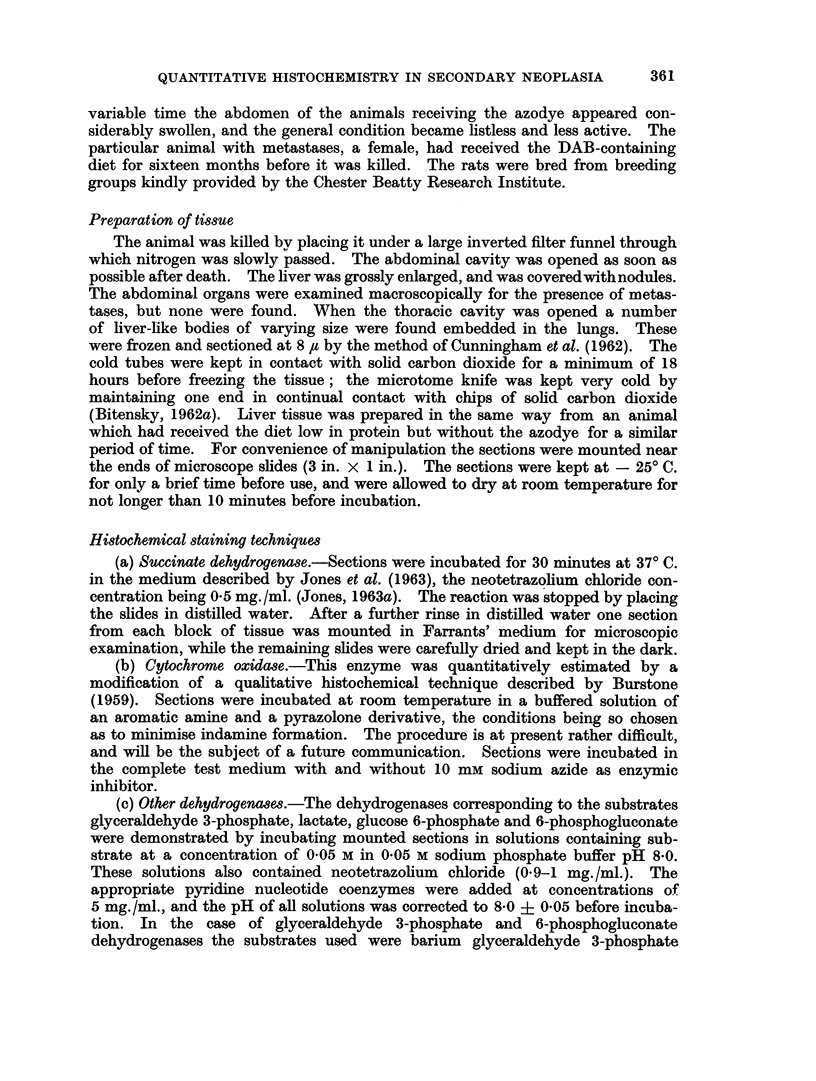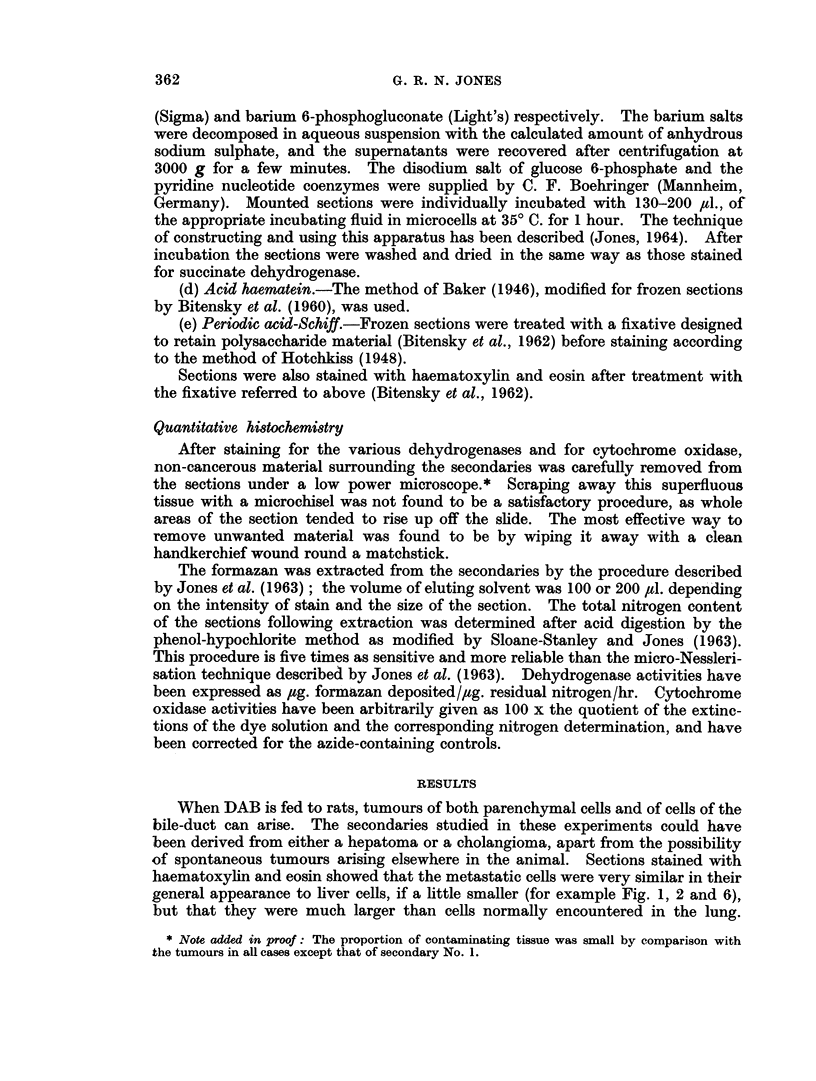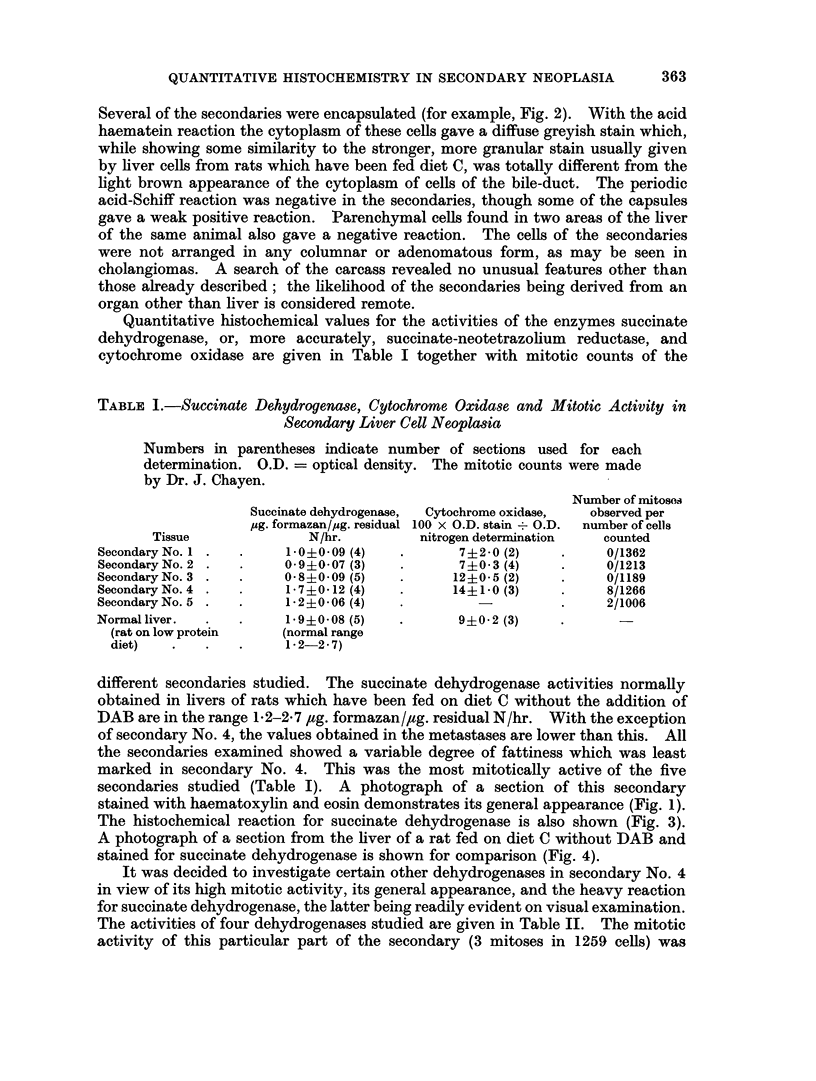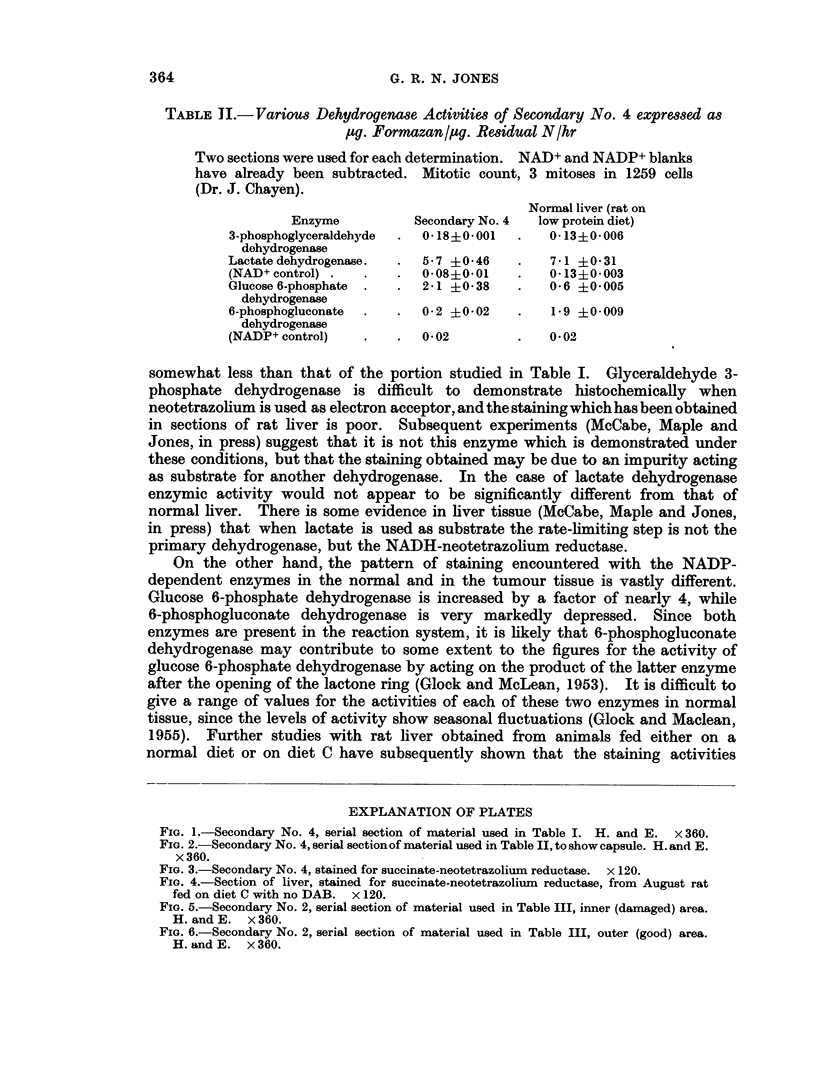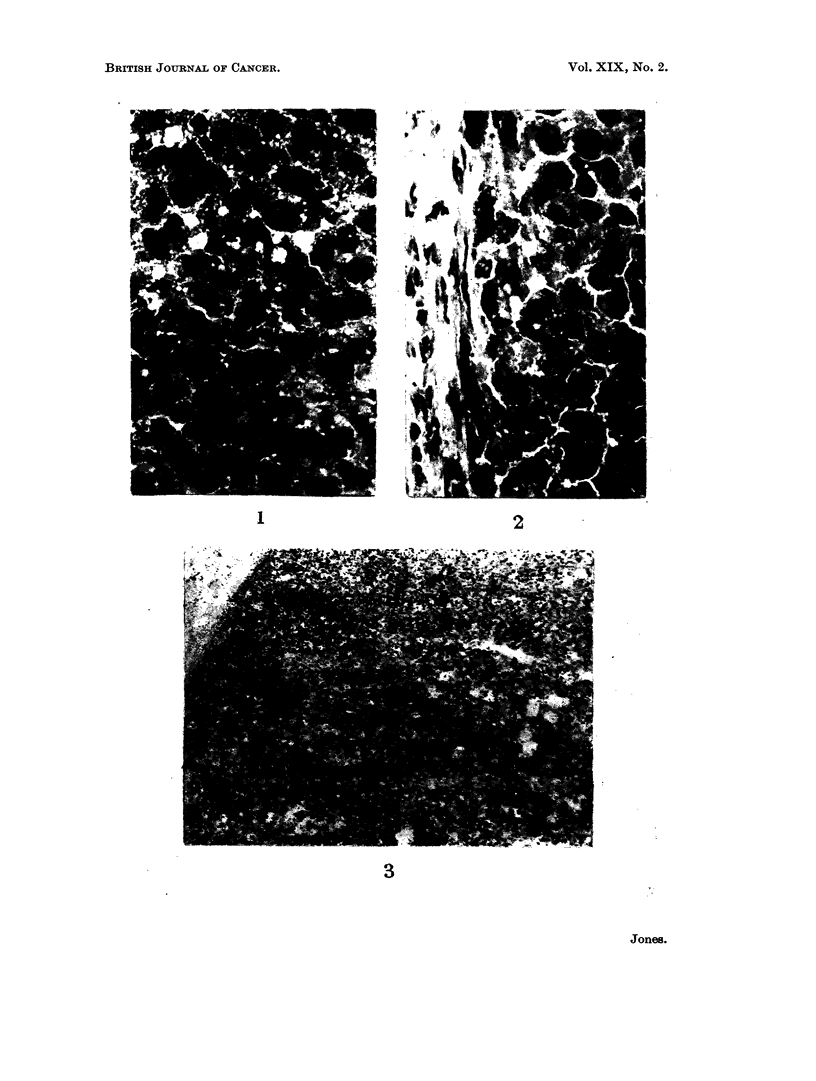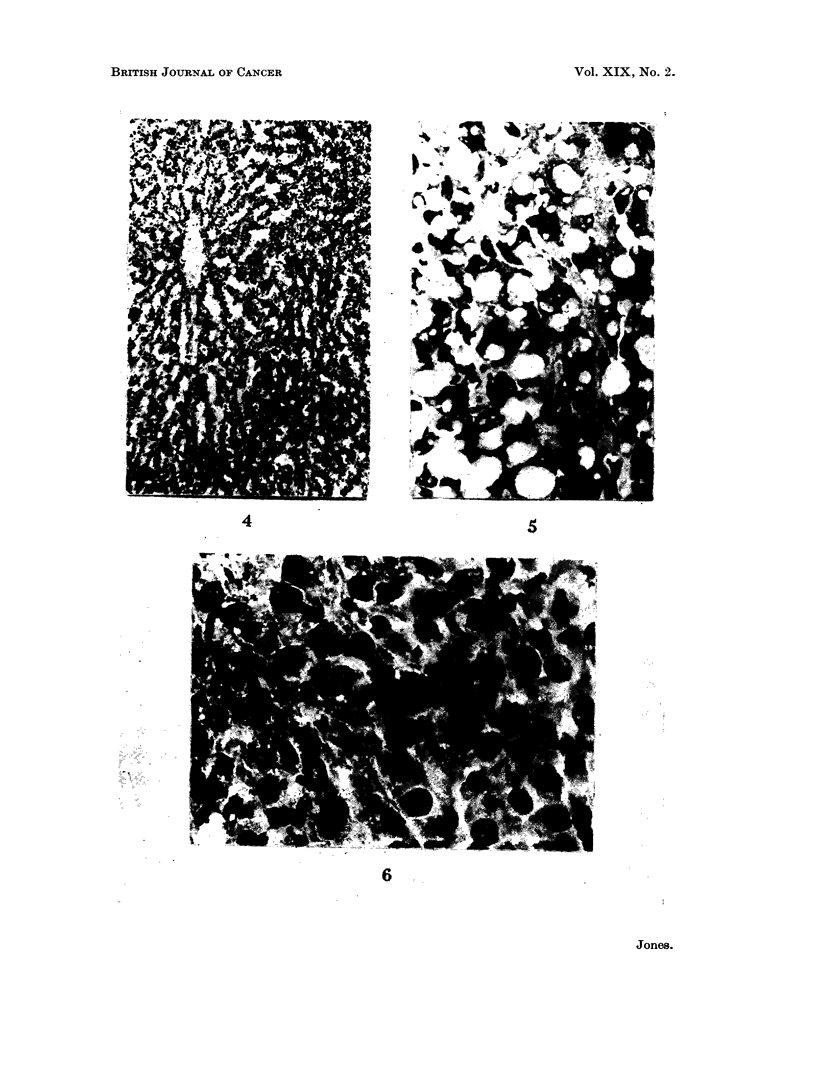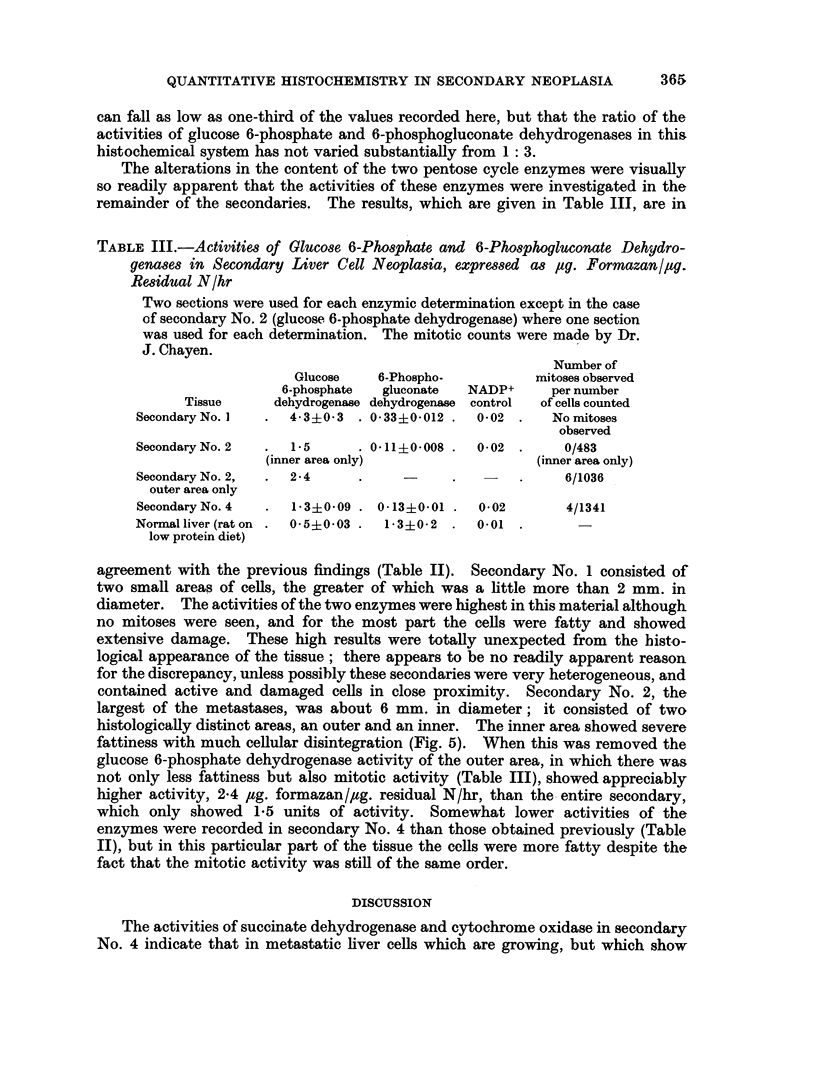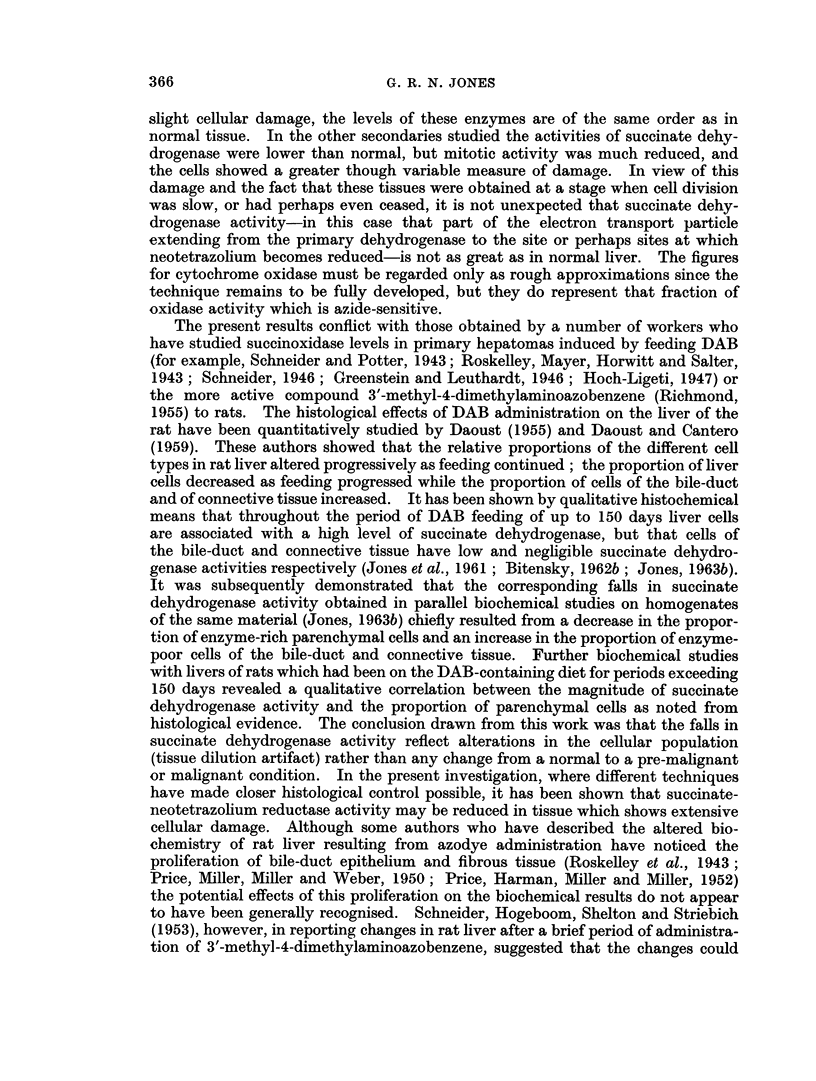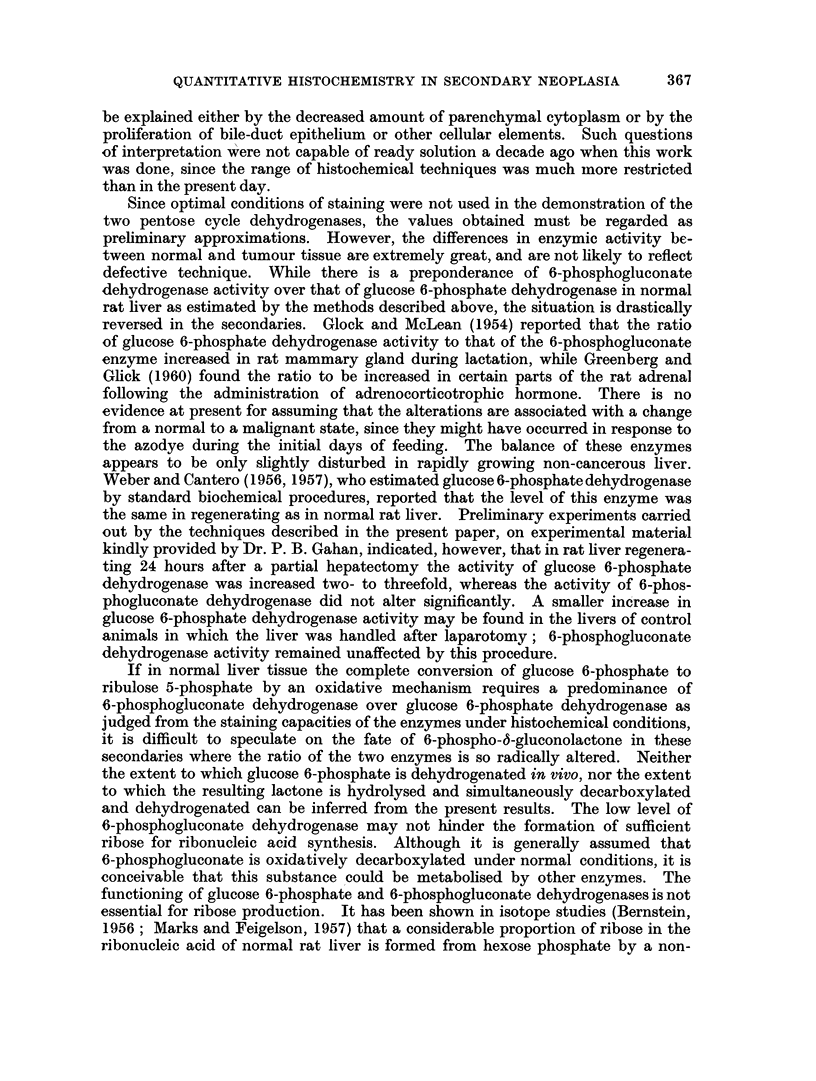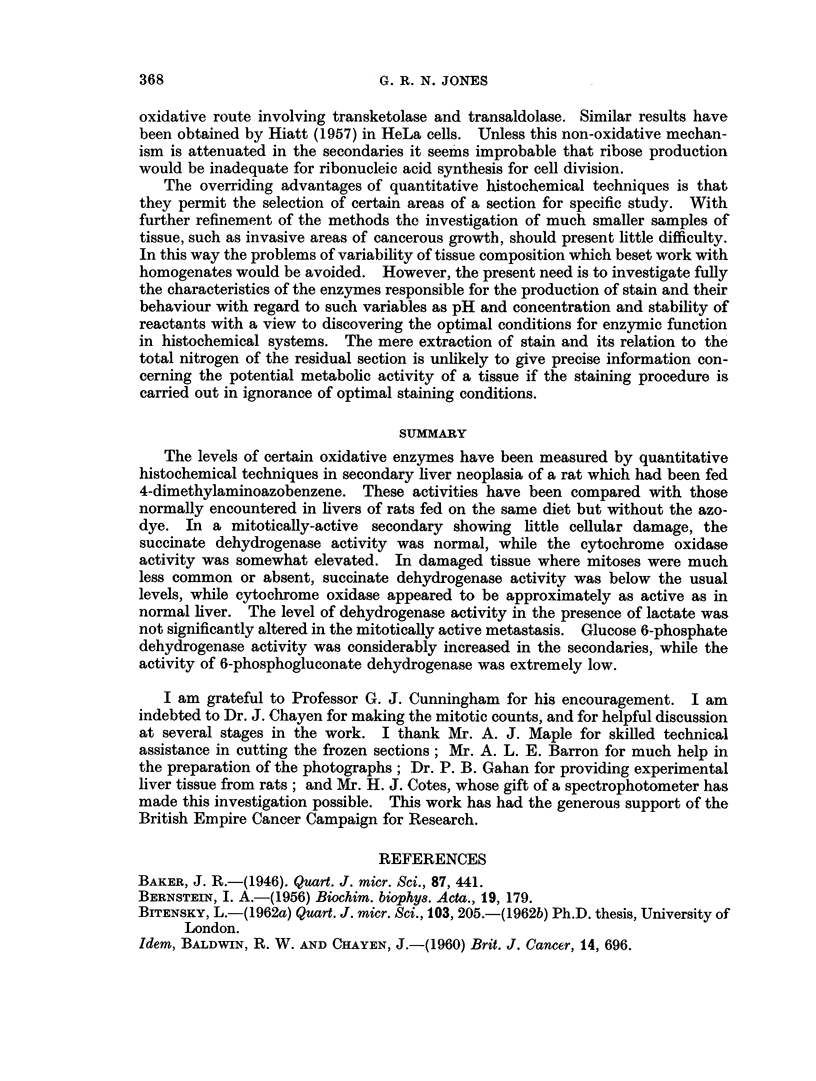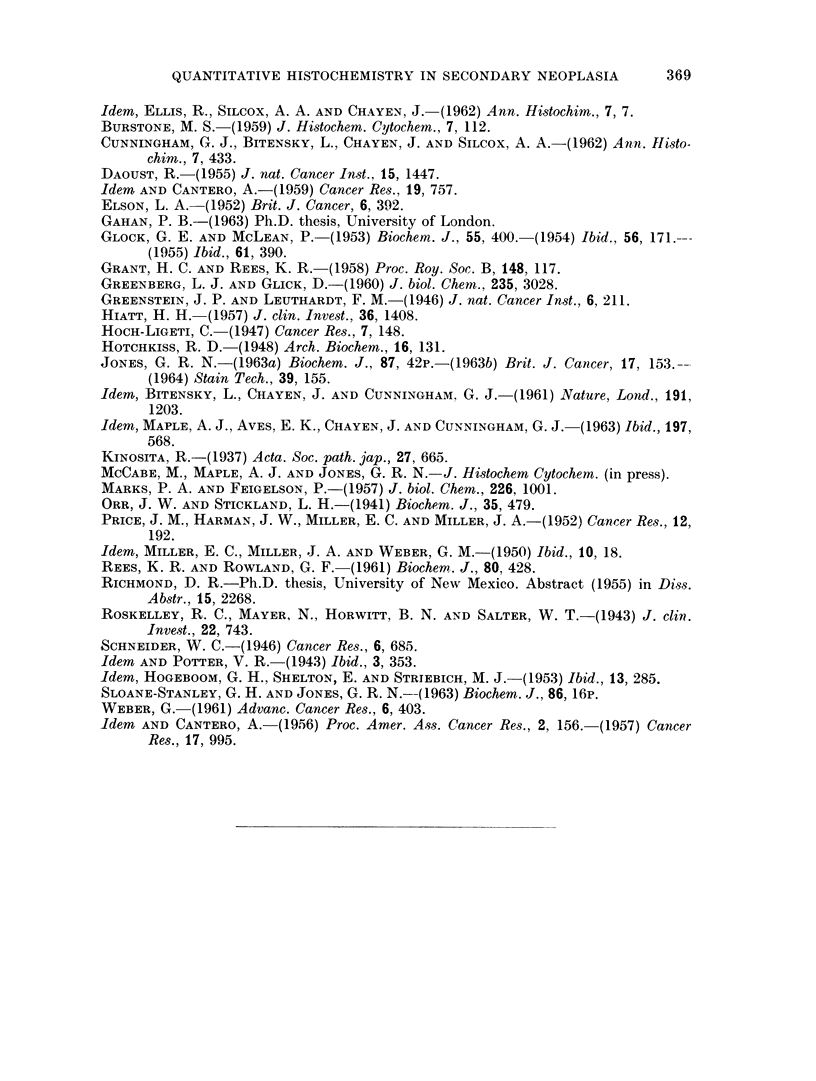# Quantitative Histochemistry: Levels of Certain Oxidative Enzymes in Secondary Neoplasia found in a Rat After Feeding 4-Dimethylaminoazobenzene

**DOI:** 10.1038/bjc.1965.43

**Published:** 1965-06

**Authors:** G. R. N. Jones

## Abstract

**Images:**


### QUANTITATIVE HISTOCHEMISTRY: LEVELS OF CERTAIN OXIDATIVE ENZYMES IN SECONDARY NEOPLASIA FOUND IN A RAT AFTER FEEDING 4-DIMETHYLAMINOAZOBENZENE

G. R. N. JONES

*From the Department of Pathology, Royal College of Surgeons of England, Lincoln's Inn Fields, London, W.C.2*

Received for publication December 4, 1964

SINCE the discovery that the administration of 4-dimethylaminoazobenzene (DAB) to rats induced liver tumours (Kinosita, 1937), this substance and certain chemically similar azodyes have been extensively used to produce such lesions for the purpose of biochemical studies. An extensive literature now exists which is concerned with comparing the biochemistry of such material with that of normal liver tissue. However, despite several histological studies in all of which the marked heterogeneity of these lesions is described, the number of investigations in which biochemical work has been histologically controlled (e.g. Orr and Stickland, 1941; Grant and Rees, 1958; Rees and Rowland, 1961; Jones, 1963*b*) remains very small. The difficulty of precise differentiation between normal and neoplastic liver cells in livers of DAB-fed rats is a considerable one, as pointed out by Weber (1961). Moreover, in cases where a mass of liver cells may show characteristics attributed to neoplasia, the problem is to distinguish such material from regenerating liver tissue. This latter shows similarities with tumour cells (Gahan, 1963), and might arise in response to the toxic effect of DAB itself. On the other hand, neoplastic cells may metastasise to other parts of the body; once metastasis has occurred, not only is the confusion of neoplasia with regeneration unlikely to be made in the secondary tissue, but also the problem of tissue dilution artifact (Jones, Bitensky, Chayen and Cunningham, 1961; Jones, 1963*b*) is avoided. With this in mind work has been started to develop quantitative histochemical techniques involving staining procedures, the techniques being sufficiently sensitive to be used as semi-micromethods for enzymic assays of secondary deposits. While many aspects of these procedures still await investigation, such methods can still give a clear advantage over classical biochemical investigations because estimations are made under histologically controlled conditions. Although the results presented here have been obtained from only one animal it is felt that both the low incidence of metastases in DAB-fed rats and the importance of the principles underlying this work justify its present publication.

### METHODS

### *Animals*

Rats of the August strain have been fed on a diet low in protein (diet C of Elson, 1952) with and without 0·06 % DAB. Tap water was freely supplied, and the diet was supplemented with 20 g. fresh cabbage three times weekly. After a variable time the abdomen of the animals receiving the azodye appeared considerably swollen, and the general condition became listless and less active. The particular animal with metastases, a female, had received the DAB-containing diet for sixteen months before it was killed. The rats were bred from breeding groups kindly provided by the Chester Beatty Research Institute.

*Preparation of tissue*

The animal was killed by placing it under a large inverted filter funnel through which nitrogen was slowly passed. The abdominal cavity was opened as soon as possible after death. The liver was grossly enlarged, and was covered with nodules. The abdominal organs were examined macroscopically for the presence of metastases, but none were found. When the thoracic cavity was opened a number of liver-like bodies of varying size were found embedded in the lungs. These were frozen and sectioned at 8 $\mu$ by the method of Cunningham *et al.* (1962). The cold tubes were kept in contact with solid carbon dioxide for a minimum of 18 hours before freezing the tissue ; the microtome knife was kept very cold by maintaining one end in continual contact with chips of solid carbon dioxide (Bitensky, 1962*a*). Liver tissue was prepared in the same way from an animal which had received the diet low in protein but without the azodye for a similar period of time. For convenience of manipulation the sections were mounted near the ends of microscope slides (3 in. $\times$ 1 in.). The sections were kept at $-$ 25° C. for only a brief time before use, and were allowed to dry at room temperature for not longer than 10 minutes before incubation.

*Histochemical staining techniques*

(a) *Succinate dehydrogenase*.—Sections were incubated for 30 minutes at 37° C. in the medium described by Jones *et al.* (1963), the neotetrazolium chloride concentration being 0·5 mg./ml. (Jones, 1963*a*). The reaction was stopped by placing the slides in distilled water. After a further rinse in distilled water one section from each block of tissue was mounted in Farrants' medium for microscopic examination, while the remaining slides were carefully dried and kept in the dark.

(b) *Cytochrome oxidase*.—This enzyme was quantitatively estimated by a modification of a qualitative histochemical technique described by Burstone (1959). Sections were incubated at room temperature in a buffered solution of an aromatic amine and a pyrazolone derivative, the conditions being so chosen as to minimise indamine formation. The procedure is at present rather difficult, and will be the subject of a future communication. Sections were incubated in the complete test medium with and without 10 mM sodium azide as enzymic inhibitor.

(c) *Other dehydrogenases*.—The dehydrogenases corresponding to the substrates glyceraldehyde 3-phosphate, lactate, glucose 6-phosphate and 6-phosphogluconate were demonstrated by incubating mounted sections in solutions containing substrate at a concentration of 0·05 M in 0·05 M sodium phosphate buffer pH 8·0. These solutions also contained neotetrazolium chloride (0·9–1 mg./ml.). The appropriate pyridine nucleotide coenzymes were added at concentrations of 5 mg./ml., and the pH of all solutions was corrected to 8·0 $\pm$ 0·05 before incubation. In the case of glyceraldehyde 3-phosphate and 6-phosphogluconate dehydrogenases the substrates used were barium glyceraldehyde 3-phosphate

(Sigma) and barium 6-phosphogluconate (Light's) respectively. The barium salts were decomposed in aqueous suspension with the calculated amount of anhydrous sodium sulphate, and the supernatants were recovered after centrifugation at 3000 g for a few minutes. The disodium salt of glucose 6-phosphate and the pyridine nucleotide coenzymes were supplied by C. F. Boehringer (Mannheim, Germany). Mounted sections were individually incubated with 130–200 $\mu$l., of the appropriate incubating fluid in microcells at 35° C. for 1 hour. The technique of constructing and using this apparatus has been described (Jones, 1964). After incubation the sections were washed and dried in the same way as those stained for succinate dehydrogenase.

(d) *Acid haematein.*—The method of Baker (1946), modified for frozen sections by Bitensky *et al.* (1960), was used.

(e) *Periodic acid-Schiff.*—Frozen sections were treated with a fixative designed to retain polysaccharide material (Bitensky *et al.*, 1962) before staining according to the method of Hotchkiss (1948).

Sections were also stained with haematoxylin and eosin after treatment with the fixative referred to above (Bitensky *et al.*, 1962).

*Quantitative histochemistry*

After staining for the various dehydrogenases and for cytochrome oxidase, non-cancerous material surrounding the secondaries was carefully removed from the sections under a low power microscope.* Scraping away this superfluous tissue with a microchisel was not found to be a satisfactory procedure, as whole areas of the section tended to rise up off the slide. The most effective way to remove unwanted material was found to be by wiping it away with a clean handkerchief wound round a matchstick.

The formazan was extracted from the secondaries by the procedure described by Jones *et al.* (1963); the volume of eluting solvent was 100 or 200 $\mu$l. depending on the intensity of stain and the size of the section. The total nitrogen content of the sections following extraction was determined after acid digestion by the phenol-hypochlorite method as modified by Sloane-Stanley and Jones (1963). This procedure is five times as sensitive and more reliable than the micro-Nesslerisation technique described by Jones *et al.* (1963). Dehydrogenase activities have been expressed as $\mu$g. formazan deposited/$\mu$g. residual nitrogen/hr. Cytochrome oxidase activities have been arbitrarily given as 100 x the quotient of the extinctions of the dye solution and the corresponding nitrogen determination, and have been corrected for the azide-containing controls.

### RESULTS

When DAB is fed to rats, tumours of both parenchymal cells and of cells of the bile-duct can arise. The secondaries studied in these experiments could have been derived from either a hepatoma or a cholangioma, apart from the possibility of spontaneous tumours arising elsewhere in the animal. Sections stained with haematoxylin and eosin showed that the metastatic cells were very similar in their general appearance to liver cells, if a little smaller (for example Fig. 1, 2 and 6), but that they were much larger than cells normally encountered in the lung.

* *Note added in proof:* The proportion of contaminating tissue was small by comparison with the tumours in all cases except that of secondary No. 1.

Several of the secondaries were encapsulated (for example, Fig. 2). With the acid haematein reaction the cytoplasm of these cells gave a diffuse greyish stain which, while showing some similarity to the stronger, more granular stain usually given by liver cells from rats which have been fed diet C, was totally different from the light brown appearance of the cytoplasm of cells of the bile-duct. The periodic acid-Schiff reaction was negative in the secondaries, though some of the capsules gave a weak positive reaction. Parenchymal cells found in two areas of the liver of the same animal also gave a negative reaction. The cells of the secondaries were not arranged in any columnar or adenomatous form, as may be seen in cholangiomas. A search of the carcass revealed no unusual features other than those already described ; the likelihood of the secondaries being derived from an organ other than liver is considered remote.

Quantitative histochemical values for the activities of the enzymes succinate dehydrogenase, or, more accurately, succinate-neotetrazolium reductase, and cytochrome oxidase are given in Table I together with mitotic counts of the

TABLE I.—*Succinate Dehydrogenase, Cytochrome Oxidase and Mitotic Activity in Secondary Liver Cell Neoplasia*

Numbers in parentheses indicate number of sections used for each determination. O.D. = optical density. The mitotic counts were made by Dr. J. Chayen.

| Tissue | Succinate dehydrogenase, μg. formazan/μg. residual N/hr. | Cytochrome oxidase, 100 × O.D. stain ÷ O.D. nitrogen determination | Number of mitoses observed per number of cells counted |
|---|---|---|---|
| Secondary No. 1 | 1·0±0·09 (4) | 7±2·0 (2) | 0/1362 |
| Secondary No. 2 | 0·9±0·07 (3) | 7±0·3 (4) | 0/1213 |
| Secondary No. 3 | 0·8±0·09 (5) | 12±0·5 (2) | 0/1189 |
| Secondary No. 4 | 1·7±0·12 (4) | 14±1·0 (3) | 8/1266 |
| Secondary No. 5 | 1·2±0·06 (4) | — | 2/1006 |
| Normal liver. (rat on low protein diet) | 1·9±0·08 (5) (normal range 1·2—2·7) | 9±0·2 (3) | — |

different secondaries studied. The succinate dehydrogenase activities normally obtained in livers of rats which have been fed on diet C without the addition of DAB are in the range 1·2–2·7 μg. formazan/μg. residual N/hr. With the exception of secondary No. 4, the values obtained in the metastases are lower than this. All the secondaries examined showed a variable degree of fattiness which was least marked in secondary No. 4. This was the most mitotically active of the five secondaries studied (Table I). A photograph of a section of this secondary stained with haematoxylin and eosin demonstrates its general appearance (Fig. 1). The histochemical reaction for succinate dehydrogenase is also shown (Fig. 3). A photograph of a section from the liver of a rat fed on diet C without DAB and stained for succinate dehydrogenase is shown for comparison (Fig. 4).

It was decided to investigate certain other dehydrogenases in secondary No. 4 in view of its high mitotic activity, its general appearance, and the heavy reaction for succinate dehydrogenase, the latter being readily evident on visual examination. The activities of four dehydrogenases studied are given in Table II. The mitotic activity of this particular part of the secondary (3 mitoses in 1259 cells) was

TABLE II.—*Various Dehydrogenase Activities of Secondary No. 4 expressed as µg. Formazan/µg. Residual N/hr*

Two sections were used for each determination. $NAD^+$ and $NADP^+$ blanks have already been subtracted. Mitotic count, 3 mitoses in 1259 cells (Dr. J. Chayen).

| Enzyme | Secondary No. 4 | Normal liver (rat on low protein diet) |
| --- | --- | --- |
| 3-phosphoglyceraldehyde dehydrogenase | $0 \cdot 18 \pm 0 \cdot 001$ | $0 \cdot 13 \pm 0 \cdot 006$ |
| Lactate dehydrogenase | $5 \cdot 7 \pm 0 \cdot 46$ | $7 \cdot 1 \pm 0 \cdot 31$ |
| ($NAD^+$ control) | $0 \cdot 08 \pm 0 \cdot 01$ | $0 \cdot 13 \pm 0 \cdot 003$ |
| Glucose 6-phosphate dehydrogenase | $2 \cdot 1 \pm 0 \cdot 38$ | $0 \cdot 6 \pm 0 \cdot 005$ |
| 6-phosphogluconate dehydrogenase | $0 \cdot 2 \pm 0 \cdot 02$ | $1 \cdot 9 \pm 0 \cdot 009$ |
| ($NADP^+$ control) | 0·02 | 0·02 |

somewhat less than that of the portion studied in Table I. Glyceraldehyde 3-phosphate dehydrogenase is difficult to demonstrate histochemically when neotetrazolium is used as electron acceptor, and the staining which has been obtained in sections of rat liver is poor. Subsequent experiments (McCabe, Maple and Jones, in press) suggest that it is not this enzyme which is demonstrated under these conditions, but that the staining obtained may be due to an impurity acting as substrate for another dehydrogenase. In the case of lactate dehydrogenase enzymic activity would not appear to be significantly different from that of normal liver. There is some evidence in liver tissue (McCabe, Maple and Jones, in press) that when lactate is used as substrate the rate-limiting step is not the primary dehydrogenase, but the NADH-neotetrazolium reductase.

On the other hand, the pattern of staining encountered with the NADP-dependent enzymes in the normal and in the tumour tissue is vastly different. Glucose 6-phosphate dehydrogenase is increased by a factor of nearly 4, while 6-phosphogluconate dehydrogenase is very markedly depressed. Since both enzymes are present in the reaction system, it is likely that 6-phosphogluconate dehydrogenase may contribute to some extent to the figures for the activity of glucose 6-phosphate dehydrogenase by acting on the product of the latter enzyme after the opening of the lactone ring (Glock and McLean, 1953). It is difficult to give a range of values for the activities of each of these two enzymes in normal tissue, since the levels of activity show seasonal fluctuations (Glock and Maclean, 1955). Further studies with rat liver obtained from animals fed either on a normal diet or on diet C have subsequently shown that the staining activities

---

EXPLANATION OF PLATES

FIG. 1.—Secondary No. 4, serial section of material used in Table I. H. and E. ×360.
FIG. 2.—Secondary No. 4, serial section of material used in Table II, to show capsule. H. and E. ×360.
FIG. 3.—Secondary No. 4, stained for succinate-neotetrazolium reductase. ×120.
FIG. 4.—Section of liver, stained for succinate-neotetrazolium reductase, from August rat fed on diet C with no DAB. ×120.
FIG. 5.—Secondary No. 2, serial section of material used in Table III, inner (damaged) area. H. and E. ×360.
FIG. 6.—Secondary No. 2, serial section of material used in Table III, outer (good) area. H. and E. ×360.

1

2

3

Jones.

4

5

6

Jones.

can fall as low as one-third of the values recorded here, but that the ratio of the activities of glucose 6-phosphate and 6-phosphogluconate dehydrogenases in this histochemical system has not varied substantially from 1 : 3.

The alterations in the content of the two pentose cycle enzymes were visually so readily apparent that the activities of these enzymes were investigated in the remainder of the secondaries. The results, which are given in Table III, are in

TABLE III.—*Activities of Glucose 6-Phosphate and 6-Phosphogluconate Dehydrogenases in Secondary Liver Cell Neoplasia, expressed as µg. Formazan/µg. Residual N/hr*

Two sections were used for each enzymic determination except in the case of secondary No. 2 (glucose 6-phosphate dehydrogenase) where one section was used for each determination. The mitotic counts were made by Dr. J. Chayen.

| Tissue | Glucose 6-phosphate dehydrogenase | 6-Phosphogluconate dehydrogenase | $NADP^+$ control | Number of mitoses observed per number of cells counted |
|---|---|---|---|---|
| Secondary No. 1 | 4·3±0·3 | 0·33±0·012 | 0·02 | No mitoses observed |
| Secondary No. 2 | 1·5 (inner area only) | 0·11±0·008 | 0·02 | 0/483 (inner area only) |
| Secondary No. 2, outer area only | 2·4 | — | — | 6/1036 |
| Secondary No. 4 | 1·3±0·09 | 0·13±0·01 | 0·02 | 4/1341 |
| Normal liver (rat on low protein diet) | 0·5±0·03 | 1·3±0·2 | 0·01 | — |

agreement with the previous findings (Table II). Secondary No. 1 consisted of two small areas of cells, the greater of which was a little more than 2 mm. in diameter. The activities of the two enzymes were highest in this material although no mitoses were seen, and for the most part the cells were fatty and showed extensive damage. These high results were totally unexpected from the histological appearance of the tissue ; there appears to be no readily apparent reason for the discrepancy, unless possibly these secondaries were very heterogeneous, and contained active and damaged cells in close proximity. Secondary No. 2, the largest of the metastases, was about 6 mm. in diameter ; it consisted of two histologically distinct areas, an outer and an inner. The inner area showed severe fattiness with much cellular disintegration (Fig. 5). When this was removed the glucose 6-phosphate dehydrogenase activity of the outer area, in which there was not only less fattiness but also mitotic activity (Table III), showed appreciably higher activity, 2·4 µg. formazan/µg. residual N/hr, than the entire secondary, which only showed 1·5 units of activity. Somewhat lower activities of the enzymes were recorded in secondary No. 4 than those obtained previously (Table II), but in this particular part of the tissue the cells were more fatty despite the fact that the mitotic activity was still of the same order.

### DISCUSSION

The activities of succinate dehydrogenase and cytochrome oxidase in secondary No. 4 indicate that in metastatic liver cells which are growing, but which show slight cellular damage, the levels of these enzymes are of the same order as in normal tissue. In the other secondaries studied the activities of succinate dehydrogenase were lower than normal, but mitotic activity was much reduced, and the cells showed a greater though variable measure of damage. In view of this damage and the fact that these tissues were obtained at a stage when cell division was slow, or had perhaps even ceased, it is not unexpected that succinate dehydrogenase activity—in this case that part of the electron transport particle extending from the primary dehydrogenase to the site or perhaps sites at which neotetrazolium becomes reduced—is not as great as in normal liver. The figures for cytochrome oxidase must be regarded only as rough approximations since the technique remains to be fully developed, but they do represent that fraction of oxidase activity which is azide-sensitive.

The present results conflict with those obtained by a number of workers who have studied succinoxidase levels in primary hepatomas induced by feeding DAB (for example, Schneider and Potter, 1943; Roskelley, Mayer, Horwitt and Salter, 1943 ; Schneider, 1946 ; Greenstein and Leuthardt, 1946 ; Hoch-Ligeti, 1947) or the more active compound 3′-methyl-4-dimethylaminoazobenzene (Richmond, 1955) to rats. The histological effects of DAB administration on the liver of the rat have been quantitatively studied by Daoust (1955) and Daoust and Cantero (1959). These authors showed that the relative proportions of the different cell types in rat liver altered progressively as feeding continued ; the proportion of liver cells decreased as feeding progressed while the proportion of cells of the bile-duct and of connective tissue increased. It has been shown by qualitative histochemical means that throughout the period of DAB feeding of up to 150 days liver cells are associated with a high level of succinate dehydrogenase, but that cells of the bile-duct and connective tissue have low and negligible succinate dehydrogenase activities respectively (Jones *et al.*, 1961 ; Bitensky, 1962*b* ; Jones, 1963*b*). It was subsequently demonstrated that the corresponding falls in succinate dehydrogenase activity obtained in parallel biochemical studies on homogenates of the same material (Jones, 1963*b*) chiefly resulted from a decrease in the proportion of enzyme-rich parenchymal cells and an increase in the proportion of enzyme-poor cells of the bile-duct and connective tissue. Further biochemical studies with livers of rats which had been on the DAB-containing diet for periods exceeding 150 days revealed a qualitative correlation between the magnitude of succinate dehydrogenase activity and the proportion of parenchymal cells as noted from histological evidence. The conclusion drawn from this work was that the falls in succinate dehydrogenase activity reflect alterations in the cellular population (tissue dilution artifact) rather than any change from a normal to a pre-malignant or malignant condition. In the present investigation, where different techniques have made closer histological control possible, it has been shown that succinate-neotetrazolium reductase activity may be reduced in tissue which shows extensive cellular damage. Although some authors who have described the altered biochemistry of rat liver resulting from azodye administration have noticed the proliferation of bile-duct epithelium and fibrous tissue (Roskelley *et al.*, 1943 ; Price, Miller, Miller and Weber, 1950 ; Price, Harman, Miller and Miller, 1952) the potential effects of this proliferation on the biochemical results do not appear to have been generally recognised. Schneider, Hogeboom, Shelton and Striebich (1953), however, in reporting changes in rat liver after a brief period of administration of 3′-methyl-4-dimethylaminoazobenzene, suggested that the changes could be explained either by the decreased amount of parenchymal cytoplasm or by the proliferation of bile-duct epithelium or other cellular elements. Such questions of interpretation were not capable of ready solution a decade ago when this work was done, since the range of histochemical techniques was much more restricted than in the present day.

Since optimal conditions of staining were not used in the demonstration of the two pentose cycle dehydrogenases, the values obtained must be regarded as preliminary approximations. However, the differences in enzymic activity between normal and tumour tissue are extremely great, and are not likely to reflect defective technique. While there is a preponderance of 6-phosphogluconate dehydrogenase activity over that of glucose 6-phosphate dehydrogenase in normal rat liver as estimated by the methods described above, the situation is drastically reversed in the secondaries. Glock and McLean (1954) reported that the ratio of glucose 6-phosphate dehydrogenase activity to that of the 6-phosphogluconate enzyme increased in rat mammary gland during lactation, while Greenberg and Glick (1960) found the ratio to be increased in certain parts of the rat adrenal following the administration of adrenocorticotrophic hormone. There is no evidence at present for assuming that the alterations are associated with a change from a normal to a malignant state, since they might have occurred in response to the azodye during the initial days of feeding. The balance of these enzymes appears to be only slightly disturbed in rapidly growing non-cancerous liver. Weber and Cantero (1956, 1957), who estimated glucose 6-phosphate dehydrogenase by standard biochemical procedures, reported that the level of this enzyme was the same in regenerating as in normal rat liver. Preliminary experiments carried out by the techniques described in the present paper, on experimental material kindly provided by Dr. P. B. Gahan, indicated, however, that in rat liver regenerating 24 hours after a partial hepatectomy the activity of glucose 6-phosphate dehydrogenase was increased two- to threefold, whereas the activity of 6-phosphogluconate dehydrogenase did not alter significantly. A smaller increase in glucose 6-phosphate dehydrogenase activity may be found in the livers of control animals in which the liver was handled after laparotomy ; 6-phosphogluconate dehydrogenase activity remained unaffected by this procedure.

If in normal liver tissue the complete conversion of glucose 6-phosphate to ribulose 5-phosphate by an oxidative mechanism requires a predominance of 6-phosphogluconate dehydrogenase over glucose 6-phosphate dehydrogenase as judged from the staining capacities of the enzymes under histochemical conditions, it is difficult to speculate on the fate of 6-phospho-$\delta$-gluconolactone in these secondaries where the ratio of the two enzymes is so radically altered. Neither the extent to which glucose 6-phosphate is dehydrogenated *in vivo*, nor the extent to which the resulting lactone is hydrolysed and simultaneously decarboxylated and dehydrogenated can be inferred from the present results. The low level of 6-phosphogluconate dehydrogenase may not hinder the formation of sufficient ribose for ribonucleic acid synthesis. Although it is generally assumed that 6-phosphogluconate is oxidatively decarboxylated under normal conditions, it is conceivable that this substance could be metabolised by other enzymes. The functioning of glucose 6-phosphate and 6-phosphogluconate dehydrogenases is not essential for ribose production. It has been shown in isotope studies (Bernstein, 1956 ; Marks and Feigelson, 1957) that a considerable proportion of ribose in the ribonucleic acid of normal rat liver is formed from hexose phosphate by a nonoxidative route involving transketolase and transaldolase. Similar results have been obtained by Hiatt (1957) in HeLa cells. Unless this non-oxidative mechanism is attenuated in the secondaries it seems improbable that ribose production would be inadequate for ribonucleic acid synthesis for cell division.

The overriding advantages of quantitative histochemical techniques is that they permit the selection of certain areas of a section for specific study. With further refinement of the methods the investigation of much smaller samples of tissue, such as invasive areas of cancerous growth, should present little difficulty. In this way the problems of variability of tissue composition which beset work with homogenates would be avoided. However, the present need is to investigate fully the characteristics of the enzymes responsible for the production of stain and their behaviour with regard to such variables as pH and concentration and stability of reactants with a view to discovering the optimal conditions for enzymic function in histochemical systems. The mere extraction of stain and its relation to the total nitrogen of the residual section is unlikely to give precise information concerning the potential metabolic activity of a tissue if the staining procedure is carried out in ignorance of optimal staining conditions.

### SUMMARY

The levels of certain oxidative enzymes have been measured by quantitative histochemical techniques in secondary liver neoplasia of a rat which had been fed 4-dimethylaminoazobenzene. These activities have been compared with those normally encountered in livers of rats fed on the same diet but without the azo-dye. In a mitotically-active secondary showing little cellular damage, the succinate dehydrogenase activity was normal, while the cytochrome oxidase activity was somewhat elevated. In damaged tissue where mitoses were much less common or absent, succinate dehydrogenase activity was below the usual levels, while cytochrome oxidase appeared to be approximately as active as in normal liver. The level of dehydrogenase activity in the presence of lactate was not significantly altered in the mitotically active metastasis. Glucose 6-phosphate dehydrogenase activity was considerably increased in the secondaries, while the activity of 6-phosphogluconate dehydrogenase was extremely low.

I am grateful to Professor G. J. Cunningham for his encouragement. I am indebted to Dr. J. Chayen for making the mitotic counts, and for helpful discussion at several stages in the work. I thank Mr. A. J. Maple for skilled technical assistance in cutting the frozen sections ; Mr. A. L. E. Barron for much help in the preparation of the photographs ; Dr. P. B. Gahan for providing experimental liver tissue from rats ; and Mr. H. J. Cotes, whose gift of a spectrophotometer has made this investigation possible. This work has had the generous support of the British Empire Cancer Campaign for Research.

### REFERENCES

BAKER, J. R.—(1946). *Quart. J. micr. Sci.*, **87**, 441.

BERNSTEIN, I. A.—(1956) *Biochim. biophys. Acta.*, **19**, 179.

BITENSKY, L.—(1962*a*) *Quart. J. micr. Sci.*, **103**, 205.—(1962*b*) Ph.D. thesis, University of London.

*Idem*, BALDWIN, R. W. AND CHAYEN, J.—(1960) *Brit. J. Cancer*, **14**, 696.

*Idem*, Ellis, R., Silcox, A. A. and Chayen, J.—(1962) *Ann. Histochim.*, **7**, 7.
Burstone, M. S.—(1959) *J. Histochem. Cytochem.*, **7**, 112.
Cunningham, G. J., Bitensky, L., Chayen, J. and Silcox, A. A.—(1962) *Ann. Histochim.*, **7**, 433.
Daoust, R.—(1955) *J. nat. Cancer Inst.*, **15**, 1447.
*Idem* and Cantero, A.—(1959) *Cancer Res.*, **19**, 757.
Elson, L. A.—(1952) *Brit. J. Cancer*, **6**, 392.
Gahan, P. B.—(1963) Ph.D. thesis, University of London.
Glock, G. E. and McLean, P.—(1953) *Biochem. J.*, **55**, 400.—(1954) *Ibid.*, **56**, 171.—(1955) *Ibid.*, **61**, 390.
Grant, H. C. and Rees, K. R.—(1958) *Proc. Roy. Soc.* B, **148**, 117.
Greenberg, L. J. and Glick, D.—(1960) *J. biol. Chem.*, **235**, 3028.
Greenstein, J. P. and Leuthardt, F. M.—(1946) *J. nat. Cancer Inst.*, **6**, 211.
Hiatt, H. H.—(1957) *J. clin. Invest.*, **36**, 1408.
Hoch-Ligeti, C.—(1947) *Cancer Res.*, **7**, 148.
Hotchkiss, R. D.—(1948) *Arch. Biochem.*, **16**, 131.
Jones, G. R. N.—(1963*a*) *Biochem. J.*, **87**, 42P.—(1963*b*) *Brit. J. Cancer*, **17**, 153.—(1964) *Stain Tech.*, **39**, 155.
*Idem*, Bitensky, L., Chayen, J. and Cunningham, G. J.—(1961) *Nature, Lond.*, **191**, 1203.
*Idem*, Maple, A. J., Aves, E. K., Chayen, J. and Cunningham, G. J.—(1963) *Ibid.*, **197**, 568.
Kinosita, R.—(1937) *Acta. Soc. path. jap.*, **27**, 665.
McCabe, M., Maple, A. J. and Jones, G. R. N.—*J. Histochem Cytochem.* (in press).
Marks, P. A. and Feigelson, P.—(1957) *J. biol. Chem.*, **226**, 1001.
Orr, J. W. and Stickland, L. H.—(1941) *Biochem. J.*, **35**, 479.
Price, J. M., Harman, J. W., Miller, E. C. and Miller, J. A.—(1952) *Cancer Res.*, **12**, 192.
*Idem*, Miller, E. C., Miller, J. A. and Weber, G. M.—(1950) *Ibid.*, **10**, 18.
Rees, K. R. and Rowland, G. F.—(1961) *Biochem. J.*, **80**, 428.
Richmond, D. R.—Ph.D. thesis, University of New Mexico. Abstract (1955) in *Diss. Abstr.*, **15**, 2268.
Roskelley, R. C., Mayer, N., Horwitt, B. N. and Salter, W. T.—(1943) *J. clin. Invest.*, **22**, 743.
Schneider, W. C.—(1946) *Cancer Res.*, **6**, 685.
*Idem* and Potter, V. R.—(1943) *Ibid.*, **3**, 353.
*Idem*, Hogeboom, G. H., Shelton, E. and Striebich, M. J.—(1953) *Ibid.*, **13**, 285.
Sloane-Stanley, G. H. and Jones, G. R. N.—(1963) *Biochem. J.*, **86**, 16P.
Weber, G.—(1961) *Advanc. Cancer Res.*, **6**, 403.
*Idem* and Cantero, A.—(1956) *Proc. Amer. Ass. Cancer Res.*, **2**, 156.—(1957) *Cancer Res.*, **17**, 995.